# Frontline healthcare workers’ experiences in implementing the TB-DM collaborative framework in Northern Ghana

**DOI:** 10.1186/s12913-021-06883-6

**Published:** 2021-08-23

**Authors:** Rita Suhuyini Salifu, Khumbulani W. Hlongwana

**Affiliations:** 1 Discipline of Public Health Medicine, School of Nursing and Public Health, University of KwaZulu-Natal, Durban, South Africa; 2 Health and Development Solutions Network, Tamale, Ghana

**Keywords:** Frontline implementers, Ghana, WHO collaborative framework, Tuberculosis-Diabetes Comorbidity

## Abstract

**Background:**

Over the past decade, global health policy has increased its focus on measures to halt further increase in tuberculosis (TB) incidence and management of diabetes mellitus (DM). However, the vertical management of these two diseases have not achieved much in addressing the adverse effects of the rising tuberculosis-diabetes co-epidemic. This necessitated the World Health Organisation and the International Union Against Tuberculosis and Lung Disease to develop a framework to manage this dual disease burden.

TB-DM co-epidemic is a public health concern in Ghana, adversely threatening the country’s fragile health systems. Since frontline healthcare workers are critical in health policy implementation, this study used Lipsky’s theoretical framework of street-level bureaucracy to explore their experiences in implementing the collaborative framework at the health facility level in Ghana.

**Methods:**

This qualitative study was conducted between July to September 2019 using an exploratory design. Data was generated using a semi-structured interview guide designed to elicit information on knowledge of TB-DM comorbidity as well as systems for co-management. Twenty-three in-depth interviews were conducted among purposively selected frontline healthcare workers (doctors, nurses, TB task- shifting officers, TB institutional coordinators and hospital managers) from three health facilities in the Northern Region of Ghana. The lead author also conducted observations and document reviews, in order to fully address the study objectives. Thematic analysis was guided by the Lipsky’s theoretical framework of street level bureaucracy.

**Results:**

The findings revealed three main themes and six sub-themes. Main themes were Prioritisation of TB/HIV co-infection while negating TB-DM comorbidity, Poor working conditions, and Coping mechanisms, whereas sub-themes were Low *knowledge and awareness of TB-DM comorbidity*, *Limited awareness of the collaborative framework*, *High workload in TB & DM Clinics, Multiple roles, Inadequate training, and Space shortage.*

**Conclusions:**

Frontline healthcare workers had limited knowledge of TB-DM comorbidity and the collaborative framework, which, in turn adversely affected the effectiveness in implementing the framework. The effective implementation of the framework begins with raising awareness about the framework through in service training amongst the frontline healthcare workers. Additionally, an integrated screening tool to detect both TB and DM would help achieve early detection of TB-DM comorbidity.

**Supplementary Information:**

The online version contains supplementary material available at 10.1186/s12913-021-06883-6.

## Background

It has been over three decades since the global acknowledgement of the connection between tuberculosis (TB) and diabetes mellitus (DM), but efforts to address the comorbidity only intensified around 2007 and 2008, owing to the rise in the TB-DM co-epidemic, worldwide [1]. In 2012, approximately 15 % of TB cases globally, were connected to DM, with China (highest populated country in the world) and India (second highest populated country in the world) alone, accounting for as high as 40 % of comorbid TB-DM [2, 3].

Individuals with DM are three times more susceptible to being infected with TB compared to their non-TB infected counterparts, pointing to the DM-induced immuno-suppression in infected patients. The projections indicate that 366 million people across the globe will be living with DM by 2030 [4, 5]. TB adversely affects individual’s glucose tolerance and leads to increased DM incidence [6]. If this trend is not averted, these projections will be a serious blow to the world’s goal of reducing TB incidence and mortality rates by 80 and 90 %, respectively, in the year 2030 [7]. The co-existence of DM and TB leads to poor treatment outcomes in both diseases [1, 8].

TB remains a serious infectious disease causing mortality and morbidity in most low-and middle- income countries (LMICs), including Ghana, with an incidence rate of 148/100,000 population per year reported in 2019 [2]. TB, a disease that is further complicated by the increasing prevalence of DM, is still a public health problem in Ghana [9]. DM prevalence among adult Ghanaians ranges from 6 to 13.9 %, with many missing cases of undiagnosed individuals in the community [10]. Estimates for TB-DM comorbidity in Ghana is limited, as reported in the study by Asante-Poku et al. [11], which discovered that DM prevalence among TB patients was two times higher than the general population. The double burden of these two diseases impose a strain on Ghana’s current health systems [12], against the backdrop of competing health priorities, such as HIV/AIDS. TB/HIV has been recognised as a public health problem in most sub-Saharan countries, including Ghana, with the World Health Organisation (WHO) classifying Ghana as a high TB/HIV co-infection burdened country [13]. Efforts to address the double burden of TB-DM resulted in the development of a collaborative framework for the care and control of TB and DM by the WHO and the International Union Against Lung Disease ( Union) [6]. This framework has set out guidelines to help national programs to adapt their health systems in order to effectively and efficiently manage patients with TB-DM comorbidity [14]. The framework makes 12 recommendations under 3 key areas; namely: (i) establishing mechanisms of collaboration, (ii) detecting and managing TB in DM patients and (iii) detecting and managing DM in TB patients [6].

Literature suggests that the successful implementation of health policies is determined by how well healthcare workers implement these policies at various levels of care, a phenomenon that can be better understood through Lipsky’s theory of street-level bureaucracy [15]. Lipsky’s theory of street-level bureaucracy (SLB) explains the attitudes and behaviour of frontline workers as they apply policy in the course of their work [15]. Lipsky’s theory is based on (i) personal discretion (ii) routines they establish and (iii) strategies frontline workers use to cope with excessive workload and uncertainties while interacting with patients [15]. Lipsky’s theory of SLB places healthcare workers at the pinnacle of the policy implementation process [16]. Therefore, healthcare workers’ understanding and perception about the policy play a pivotal role in influencing how and which aspects of the policy gets or does not get implemented, including the implementation modifications thereof [16]. Frontline healthcare workers do interact with patients daily in the course of service delivery, hence it is during this interaction that they use the resources available to them and their discretion to “re-make” the policy, based on their subjective assessment of the situation [16–18].

## Methods

### Study setting

Data collection for this study took place from July 2019 to September 2019, in the Northern region (NR) of Ghana. Ghana is a West African country with an estimated population of over 28 million people [19]. The country shares borders with Togo in the east, Ivory Coast in the west and the Burkina Faso in the north, all of which are French speaking countries and the Gulf of Guinea to the south [20]. The NR is one of the 16 regions of Ghana, with a population 1,905,628 and the capital Tamale is the 3rd largest urban centre in the country [19]. This study took place in 3 purposively selected hospitals, as they all offer both DM and TB services at the same facility.

### Study design

This exploratory qualitative study was aimed at understanding the experiences of frontline healthcare workers in implementing the TB-DM collaborative framework in Ghana [21]. Healthcare workers were drawn from TB and DM units in the selected facilities, using heterogenous purposive sampling techniques. This approach allowed us to recruit diverse participants with respect to roles, age and gender, in order to obtain multiple perspectives on the phenomenon.

#### Ghana health system and theoretical framework

**Fig. 1:**
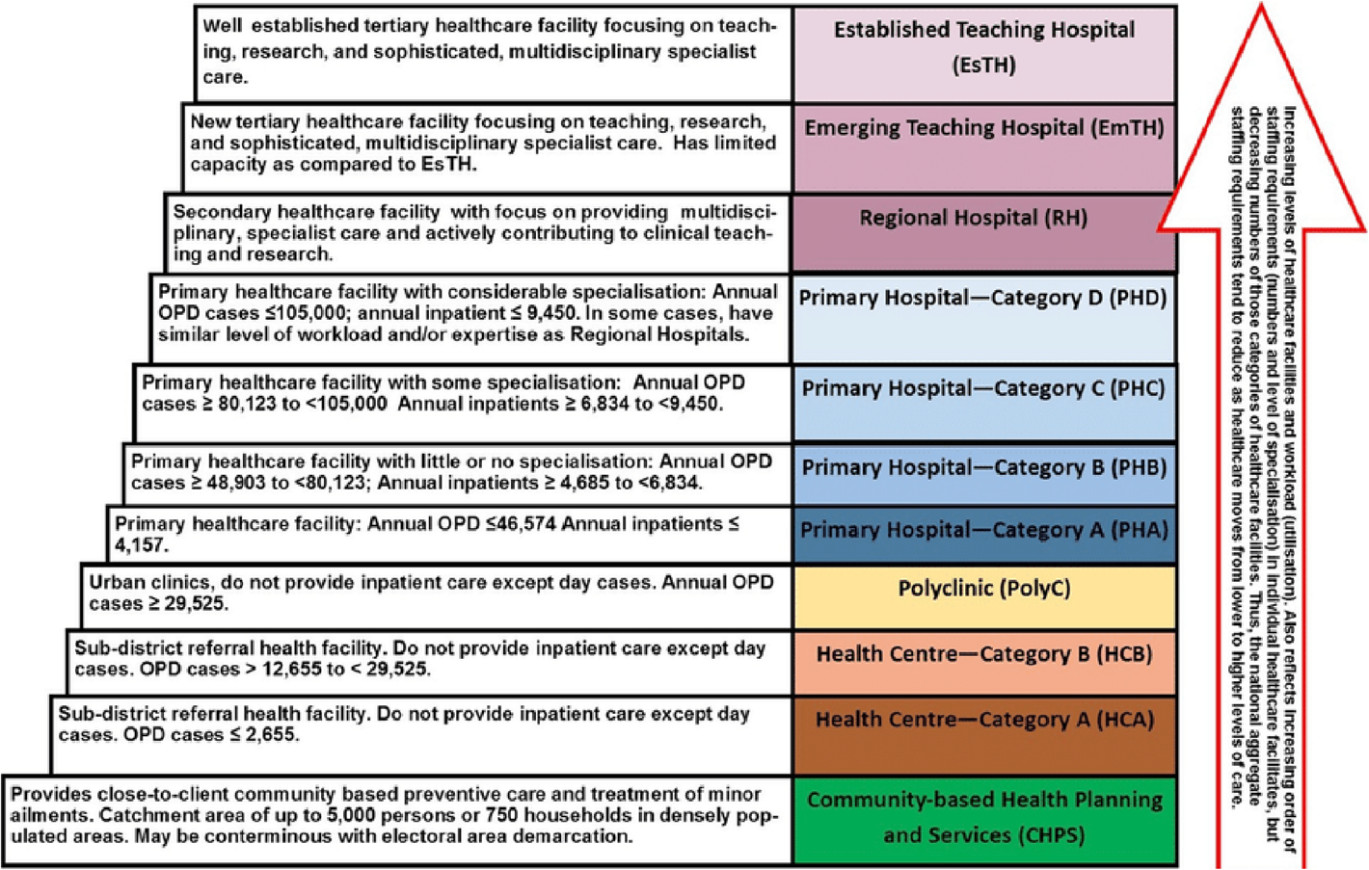
Categorisation of Ghana Health Care System. Source: Forecast of Healthcare Facilities and Health Workforce Requirements for the Public Sector in Ghana, 2016–2020) [23]

While it is important to understand the application of Lipsky’s theory of SLB in the implementation of TB-DM collaborative framework in Ghana, this should be done within the context of full appreciation of the country’s health care system. Ghana’s healthcare system is comprised of both public and private health facilities, which are divided into three levels of care, namely: primary, secondary and tertiary levels [22]. Health services delivered at primary level of care cover community based health planning services compounds (CHPS), health centres, clinics and hospitals, which serve community, subdistricts and districts (Fig. 1) [20, 22]. Secondary care is offered at the regional level, and teaching hospitals offer tertiary care and training of healthcare workers (Fig. 1) [20, 22, 23].

The multi-level healthcare system in Ghana has Teaching hospitals, Regional hospitals, Primary hospitals at the district level and health centres at the sub-district [23]. At the national level, teaching hospitals are set up to receive referrals of complicated health conditions from all over the region or country, which cannot be handled at the other levels of care. These teaching hospitals are by design linked to universities in order to improve service delivery [23]. Regional hospitals offer specialised healthcare, and they are a referral point for health conditions from the district hospitals. Sub-districts are equipped to manage basic preventive and curative health conditions [23].

The aim of this study was to explore the frontline healthcare workers’ experiences in implementing the TB-DM collaborative framework, using the bottom-up approach, including how they understand their roles as front liners and the negotiations they make in the course of implementation. This study took place at different levels of care, but these facilities have been hidden to protect the anonymity and confidentiality of the participants.

### Data generation

Data were generated using in-depth interviews, observations, and document reviews. In-depth interviews((Supplementary files 1 and 2) were conducted by the lead author with twenty-three healthcare workers purposively selected from three health facilities. The main stakeholders interviewed were doctors (*n* = 3), nurses (*n* = 12), institutional coordinators (*n* = 3), task shifting officers (*n* = 3), and hospital managers (*n* = 2) (Table 1). The lead author spent time at the various health facilities during the clinic days to conduct nonparticipant observation of the clinic sessions. The lead author also reviewed a number of documents related to care and management of TB and DM. The reviewed documents included screening tools, manuals, enrolment forms, patients registers and NTP annual reports.

### Data analysis

Twenty-one in-depth interviews conducted in English for a duration of 45 min to 1h were audio-recorded (with participants’ permission). Two respondents (institutional coordinator and medical doctor) declined to have interviews audio-recorded, but field notes were taken during the interviews. Audio recordings were transcribed verbatim, and the field notebook was used to record observations. Transcripts were primarily reviewed by the lead author prior to being shared with the second author for quality check and verification. Similar responses were coded and grouped into main and sub-themes. Observation and document review provided useful information for the better understanding of the contexts in which healthcare workers performed their duties. Analysis was guided by Lipsky’s framework, focusing on personal discretion, routines established, and strategies adopted to cope with high workload and scarce resources. This approach proved helpful to gaining further insights on how frontline healthcare workers’ day-to-day activities affect and/or are affected by the health policy implementation.

This study obtained ethical approval from both the University of KwaZulu-Natal (UKZN) Biomedical Research Ethics Committee (BREC) (Reference Number: BE262/19) and Ghana Health Service Ethics Review Committee (Reference Number: GHS_ERC 012/04/19). The Northern regional health directorate sent formal letters about the research to the managers of the three selected hospitals. Health facility managers gave the researcher permission to conduct the research. Selected healthcare workers in the TB and DM units who met the criteria and were willing to participate in the study were recruited. The participants were also reassured that they could opt out of the interview process at any time if they were uncomfortable and that would not affect them in anyway whatsoever. All study participants read the information sheet and signed the informed consent forms. Interviews were conducted at the participants’ preferred private locations, with limited interruptions, to ensure they were comfortable to share. To ensure the credibility of data generated from the interviews, the lead author used the member check technique, through repeating the answers to participants to allow them to clarify or correct any errors in their response. This was further strengthened by supporting the findings produced through the analysis with verbatim quotes.

## Results

Twenty-three healthcare workers from the 3 purposively selected health facilities with TB and DM units participated in this study. These comprised of 12 Nurses, 3 Task-shifting Officers, 3 Medical Doctors, 3 TB Institutional Coordinators and 2 Hospital Managers. The work experience of participants ranged from 5 to 35 years with the mean experience of 14 years and gender distribution of fourteen males and nine females (Table 1).

**Table 1:** Sociodemographic profile of frontline healthcare workers

Role	Gender	Years of experience	Educational level
Nurse-TB care	F	11	Dip. Nursing, BSc Nutrition
Nurse-TB care	M	9	Dip. Nursing
Nurse-TB care	M	9	Dip. Nursing
Nurse-TB care	F	25	Dip. Nursing
Nurse-TB care	F	9	BSc Nursing
Nurse-TB care	M	5	BSc Nursing, MPH
Nurse-DM care	F	9	Dip. Nursing
Nurse-DM care	F	6	Dip. Nursing
Nurse-DM care	F	5	Dip. Health assistant
Nurse-DM care	M	12	BSc Nursing, MA Clinical leadership
Nurse-DM care (AD)	M	8	Dip. Nursing
Nurse Prescriber-DM care	M	17	BSc. Nursing
Task shifting officer-TB care	F	7	Dip. Nursing
Task shifting officer-TB care	M	5	BSc. Community Development
Task shifting officer-TB care	F	10	Dip. Management
Medical Doctor -DM care	M	35	MB ChB
Medical Doctor -TB care	M	10	MB ChB, MPH
Medical Doctor-DM care	M	8	MB ChB
Institutional coordinator-TB care	M	9	PharmD
Institutional coordinator-TB care	F	35	Dip. Nursing, Midwifery
Institutional coordinator-TB care	M	33	Dip. Comm. Disease, BSc. Pub.Health
Hospital manager/Medical Doctor	M	23	MB ChB
Hospital manager/Medical Doctor	M	26	MB ChB

This research revealed three major themes and six sub-themes pertaining to the frontline healthcare workers’ experiences and these themes/ sub-themes aligned well with Lipsky’s explanation on how frontline healthcare workers translate policy in their day-to-day practice [15]. These themes were: (1) Prioritisation of TB/HIV co-infection while negating TB-DM Comorbidity, (*2*) Poor working conditions, and (3) Coping mechanisms, with the emergent sub-themes being: (1) Low knowledge and awareness on TB-DM comorbidity, (2) Limited awareness of the collaborative framework among healthcare workers, (3) High workload in TB & DM clinics, (4) Multiple roles, (5) Inadequate training, and (6) Space shortage (Table 2).

Healthcare workers shared their experiences in implementing the collaborative framework in their arguably challenging work environment, navigating dual roles with inadequate training to be competent in both. However, in order to cope with the dual roles, these healthcare workers initiated self-funded activities and community visits, in order to continue with the delivery of healthcare to their patients in line with the provisions of the policy.

**Table 2:** Summary of Themes and Sub-themes

Theme	Sub theme
**Prioritisation of TB/HIV co-infection while negating TB-DM comorbidity**	Low knowledge and awareness on TB-DM comorbidity
	Limited awareness of collaborative framework.
**Poor working conditions**	High workload in TB & DM Clinics
	Multiple roles
	Inadequate training
	Space shortage
**Coping mechanisms**	

### Prioritisation of TB/HIV co-infection while negating TB-DM Comorbidity

Ghana has a high burden of TB and HIV/AIDS co-infection with a well-established system, covering the continuum of care for patients with TB/HIV co-infection, as observed during the implementation of this study in the Northern Region. There exist clear guidelines on screening and referrals, as well as monitoring mechanisms between the TB unit and the HIV/AIDS unit. These structures guide health workers in managing patients with dual infection. Interview of frontline healthcare workers participating in this research revealed that they were more knowledgeable about the management of TB/HIV co-infection, compared to TB-DM comorbidity. One frontline healthcare worker shared her view as follows:

*When the patient is positive for TB, we also do HIV screening. The HIV unit also does screening for TB. They have our [TB] screening tool and when they have an HIV positive patient, they also run the test. We refer their cases [HIV-positive] there and they also refer our cases [TB positive] here.”***Female-TB task shifting officer**.

“… *the HIV /AIDS guideline says you test anyone who has TB for HIV but there is no information on testing TB patients for the diabetes*.” **Male TB institutional coordinator**.

*“In this facility there is no collaboration between the diabetes clinic and TB unit.”****Male-DM care nurse***.

*“The two programs, namely: HIV /AIDS and TB have a better collaboration, where anybody who is HIV positive is screened for TB and anybody who is TB positive is screened for HIV because of the high frequency of the two conditions existing together.****”*Male-Hospital Manager**.

“*It’s a routine that any client you put on TB treatment you test for HIV. At the HIV clinic they are also given the TB kits because people living with HIV are prone to TB. likewise, they also leave the HIV test kit here for us to test TB patients*.” **Male -TB care nurse**.

### Low knowledge and awareness on TB-DM comorbidity

This research found that there was low knowledge about TB-DM comorbidity among healthcare workers in the three facilities, which may inadvertently contribute to it being profiled as low priority. The researcher observed that staff working with TB patients exhibited signs of being more aware of the comorbidity than DM staff. They appeared more confident and shared more information on TB-DM comorbidity than their counterparts in the DM unit. Most of the participants did not know about TB-DM comorbidity. and had not encountered a patient with the comorbidity.

“*For now, we don’t have any information about the TB-Dm comorbidity.*” **Male-DM care Nurse**.

*“…… I have not seen a client with the TB-DM comorbidity.”****Male-DM care nurse***.

### Limited awareness of collaborative framework

The framework outlines strategies for bidirectional screening of TB and DM patients, as well as the collaboration between the two programs (TB and DM). However, this study found low awareness of the framework by the frontline healthcare workers, which compromised their full appreciation of the true value of all the components of the framework, including the objectives it aims to achieve.

*“… it’s just recently we heard of it [TB-DM], someone doing a research into TB-diabetes comorbidity shared with us about this just about a month ago. We have not yet encountered anyone with the comorbidity. Because the work is so much, sometimes we even forget to check and there is no place in the form we fill to check for diabetes.”* **Female- TB care institutional coordinator**

Screening for TB among patients in the three facilities participating in this research was more widely known than screening for DM.

“*What we want to make sure is that nobody comes to the hospital and goes without being screened for TB, hence the emphasis on TB screenin*g.” **Male-TB care medical doctor.**

“*For the diabetic side we don’t have any kit to test our TB patients for diabetes. We have sent the screening tool and lab request form and sputum containers to the diabetic clinic. With the diabetic clinic we normally give them our screening tools to screen clients suspected of TB*”. **Male-TB care nurse**.

The review of documents from the TB units also showed clear guidelines on screening DM patients for TB. This was particularly evident in, symptom-based screening tool, sputum examination request form, charts, TB treatment card, and reports. However, there were no clear guidelines on how to test the TB patients for DM at the TB departments visited. Healthcare worker however agreed that there was a need to know more about this rising comorbidity and were open to getting more information.

*I have heard that someone with diabetes can have TB. The TB unit should organize more workshops for us [DM care], so we can know more*.” **Female-DM care nurse**.

#### Poor working conditions

Respondents in this study expressed concerns about factors that hindered their effectiveness in service delivery, mainly the working conditions and how this impacted on the manner in which they performed their roles of caring for TB and DM patients. These were high workload, being involved in playing multiple roles, inadequate training, and space shortage.

##### High workloads in TB & DM Clinics

The gravity of the current workload expressed by healthcare workers appeared to influence their reluctance to take on TB-DM co-management as an additional function. This concern was shared by all healthcare workers offering DM and TB in the facilities that participated in this study. Routine DM care in all the facilities was only available for two days in a week, leading to high work volumes, and this was considered inadequate.

*“… we see averagely 30 or 35 patients but today we have 50 patients my head is even aching. I came as early as 7am, there is so much pressure and you can’t leave till the last patient leaves the pharmacy because if they don’t get the medication you need to rewrite the medication and those using insulin for the first time have to come back for training on how to use it.”***Female-DM care nurse**.

Newly diagnosed patients and patients who already had diabetes came for care and regular review only on the given days with less flexibility for care outside the stipulated days. The researcher observed this situation during time she spent at the facility on clinic days, reviewing the registration book and treatment guidelines, as well as when respondents shared on their work routines.

### Multiple roles

Responses from some participants and the researcher’s own observations corroborated data on the multiple roles played by the healthcare workers. Healthcare workers in some health facilities had to perform multiple roles in addition to delivering TB or DM services, as expressed by the respondents in the quotes below:

*The work is a lot, you saw the number of people at the OPD. I am at the OPD, there are other roles I had to pick up. I have learnt how to take vital signs, whilst I am helping the nurses, I can also screen the patients for TB.”***Male-TB Task-shifting officer**.

*“Presently one nurse has to manage the patient with both diseases, the one they came reporting for and the TB detected in the course of testing, that is also a challenge.****”*Female-TB care nurse**.

***“****Staffing is a big issue; we were three [staff] and one went on retirement and one left for school [studying], so I am the only prescriber [now]. We are hoping others will join.”***Male-DM care nurse**.

“*One of the challenges in TB, is human resources. Apart from care for the patients, there is so much reports to write and data to enter, it makes the work tedious.”***Female-TB care institutional coordinator**.

“*We close clinical work around 4:30. I am just coming from there, then I come to the office to do administrative work. I was about climbing the steps when you called me. The number of doctors you know is low and it’s the rainy season, malaria etc., the facility is supposed to run 24 hours emergency and other things and you have two people running emergency, so I try to get myself involved.”***Male-hospital manager**.

### Inadequate training

Healthcare workers did not feel adequately equipped to handle cases of TB-DM comorbidity, as they, reportedly, neither been trained or had received inadequate training. DM healthcare workers had not been trained in managing this comorbidity, and on the TB side, either very few trainings had been provided or trainings had insufficient depth. Healthcare workers felt that training on the guidelines for co-management of TB and DM was woefully inadequate and when conducted, it was irregular and far in between. Training for TB staff was mostly organised from the Ghana National TB control program. One respondent noted:

*“…We don’t normally train with other departments, mostly trainings are longitudinal from the National TB control program.”***Female-TB care nurse**.

*No training since I came- I have never attended any TB workshop not one. How will I know more about the TB?***Female-TB task-shifting officer**.

*“The other thing has to do with the staffing and skills training, because all of us are general nurses. After a year or two you [staff] are sent to a specialized place to work, without necessary giving you any training to handle such conditions so you learn on the job. Maybe the senior [staff] you meet will take you through. …. these are some of the challenges*.***” Female*-DM care nurse**.

***“****I went for a TB training, but it’s been a while more than 4 years ago. I recommend that more in-service trainings should be given to staff at the facility level, because medicine is dynamic and day-in day out there are updates.****” Female*-TB care nurse**.

Retirement and transfers also affected the retention of knowledge gained through trainings.

*“No joint training that I can remember. We used to have the training but there were changes and they took those nurses out of here and two people went on retirement. About five staff have gone away they have not been replaced.”***Female-DM care nurse**.

#### Space shortage

Respondents identified infrastructural challenges as constraining factors to the delivery of services to DM and TB patients. The lack of permanent space dedicated to DM clinics forced healthcare workers to restrict patient care to only 2 days a week.

*“… at the moment our main problem is space, we don’t have a place of our own so we hold our clinics only twice in a week and that makes it very tiring because we have a whole lot of patients who will come only on Tuesdays and Fridays. Sometimes it makes it difficult to give them the necessary care they need.”* **Female-DM care nurse.**

“*We are lobbying for a permanent place so patients can come every day. So, the number of patients will also reduce per day.*” **Male-DM care nurse**.

In one health facility the TB unit had to share the same space with HIV unit, which may have an effect on the stigmatisation of the patients and also negatively affected the strict adherence to confidentiality and privacy protocols during the screening and treatment.

*“Many patients don’t like to be screened they are afraid of the condition TB. Even staff are hesitant to be screened. The TB people take their drugs with the ART people in one room, so anyone seen coming from the room is suspected to have HIV.”***Male-DM care nurse**.

The respondents also shared the challenges faced during counselling sessions with TB patients in congested consulting rooms with patients with other conditions. The researcher observed them trying to be discreet by speaking in low tones to prevent the other patients from hearing the discussion.

*“Space is our challenge, this room is not a TB unit alone, we do PMTC, give ART services here, and dispense drugs for both TB and ART. There is not enough space to see all these patients.”***Female-TB care institutional coordinator**.

*“Secondly, human resource is a problem; we are the only two [staff] in this room handling all the services I have mentioned and managing the units.*” **Female-TB care nurse**.

*“We have a challenge with space, we don’t have a specific ward for TB patients. We have only one room for isolation of TB patient with only one bed and the room is for both male and female. So, if a female patient is there and a male patient is diagnosed with TB, you can imagine what will happen. So, one is forced to put patients in the general ward where you nurse with other patients.” ****Female*-DM care nurse**.

“*We need a bigger place because privacy is key in our management. Some of the patients fail to come to the clinic because they come and sit in an open place and we do our education together. Patients prefer to be seen as individuals but because of the space we are not able to do so. If we are given a larger place, we can partition, and each nurse will have a place and invite the patient to sit and discus his or her condition.”***Female-DM care nurse**.

### Coping mechanisms

Healthcare workers developed coping mechanism by using their “discretion” to find ways to “cope” with the challenges presented in this paper to deliver care, despite the challenging work environment and scarce resources. Some of these initiatives were follow-ups to patients’ homes by hospital staff and supporting patients with money for transportation.

*“… contact tracing for everybody is based on my own initiative. We are supposed to go out, but we have not been given the resources, so I go on my own. I go to the patients’ houses. If I look at the patients’ area and it’s in town I just go to their house when I’m coming to work. I leave very early and pass by their home talk to the family get a few samples from the family members.”****Male-TB task-shifting officer***.

*“Sometimes the patient default because of money for transportation, sometimes we take from our own pockets. We need some funding to support the patients who cannot afford to come to the hospital*.” **Female-TB care nurse**.

“*The hospital does not have the resources for transportation. Sometimes I have to use my personal money to trace and follow-up on patients. We don’t have that support. If we can get those things, I know we can detect more cases because I know TB now is back.”***Female-TB task shifting officer**.

## Discussion

This research explored healthcare workers’ experiences in implementing the collaborative framework at three selected health facilities in Ghana guided by Lipsky theory of street level bureaucracy. Three main themes and six sub-themes emerged from the data analysis and these were: (I) Prioritisation of TB/HIV co-infection while negating TB-DM comorbidity (Low *knowledge and awareness of TB-DM comorbidity*, Limited awareness of the collaborative framework) (II) Poor working conditions *(High workload in TB & DM Clinics, Multiple roles, Inadequate training and Space shortage)* (III) Coping mechanisms. The manner in which frontline healthcare workers managed high workloads, handled space shortage, navigated multiple tasks and coped with the challenges, aligned well with Lipsky’s theory. For example, frontline healthcare workers restricted patient care to only 2 days a week, leading to the high workloads owing to a lack of permanent space dedicated to DM clinics. These frontline healthcare workers also learned to perform multiple roles all at once. For these frontline healthcare workers, coping mechanisms came with personal sacrifice, both physically and financially. For example, frontline healthcare workers have had to use their personal monies to trace and follow-up on patients. All these actions by the frontline healthcare workers directly talk to Lipsky’s theory of street-level bureaucracy.

Apart from the aspects of the results that align with Lipsky’s theory, these result of this study are also consistent with literature, which has shown that a challenge posed by HIV/AIDS to TB control, has led to many countries efficiently implementing policies to manage TB/HIV coinfections [1, 13]. Our research found that staff were well versed with Ghana’s policy on TB/HIV integration than they were with the TB/DM collaborative framework [13]. The observations in this study, showed screening TB patients for HIV/AIDS and vice versa was routinised and its implementation can provide key lessons to guide effective implementation of the WHO–Union collaborative framework. This could be explained by Lipsky’s theory which argues that the success of a policy is largely dependent on how well the healthcare worker understands it and communicates to the citizens [15].

In this study TB healthcare workers knew more about the policy to screen DM patients for TB and were better equipped but had little or no knowledge about screening TB patients for DM. Similar findings were reported in studies from Nigeria and India [24–26]. DM health care workers were mostly not aware of the policy and the need for bidirectional screening. This may be as a result of healthcare workers having little or no knowledge of the comorbidity and the devastating effect of TB on DM and vice versa, as supported by a few studies [26–28]. Frontline healthcare workers therefore were not well equipped to handle the TB-DM comorbidity, the same situation was reported in other studies [24, 27, 28]. The findings highlight that limited trainings were organised on bidirectional screening, hence healthcare workers were not adequately equipped for the additional role of detecting and managing patients with the dual condition of TB and DM [29]. Consistent training of healthcare workers caring for TB and DM patients is anticipated to yield better results in identifying TB-DM comorbidity and early care and control, as demonstrated by Agarwal et al. [30], where training of healthcare workers and established infrastructure resulted in significant improvements in service delivery .

This study revealed high workload in TB and DM clinics, which was consistent with findings in Mexico [31]. This situation was further exacerbated by staff going on retirement, study leave and transfers similar to other studies [26, 31]. The pressure of high workload and limited space allocation found in this study gave rise to healthcare workers having to perform multiple roles. This was how they managed and coped with the high workload [32]. However, healthcare workers found this situation taxing and overburdening, which differed from the findings of some studies, where healthcare workers embraced other duties as growth opportunity [29, 33].

In this study healthcare workers caring for TB and DM patients faced a number of challenges in implementing the collaborative framework at the health facility level. Consistent with Lipsky’s theory of street level bureaucracy [18], the healthcare workers, in spite of these challenges, also had developed innovative ways to achieve unhindered delivery of care services to their patients. The initiatives of healthcare workers in this study was to self-finance in order to conduct follow up visits to patients, an approach that differed from a Nigerian study, where some healthcare workers collected gifts and informal payments from patients or even asked for a fee for home visits [34]. However, the follow-up done in this study are likely to have favoured patients living in nearby urban areas, as opposed to those in the distant countryside, given that home visits made by the healthcare workers were self-financed and within the travel routes of healthcare workers. One study found healthcare workers’ coping strategy to be positive attitude [26], this was not explicit in our study, but the actions of the healthcare workers showed their positive attitude and commitment.

### Limitations and recommendations

The limitations of the study were two-fold, namely: non-inclusion of health facilities from the private sector and non-participation of community-based health planning services compounds (CHPS).

These limitations are important given that the perspectives from the study participants miss insights from private sector frontline healthcare workers and CHPS compounds.

Despite these limitations, we considered that the impact of the policy under review is more pronounced among the frontline healthcare workers in public health facilities, as they receive high volume of patients requiring TB and DM care services, compared to their private sector counterparts. Secondly, the public health sector employs a large proportion of frontline healthcare workers, hence any improvements in their implementation of the TB-DM collaborative framework are likely to make a real difference in reducing TB-DM morbidity and mortality. Therefore, while the incorporation of private for-profit health facilities may have added a unique perspective, this does not affect the utility of the results.

The CHPS compounds is Ghana’s primary healthcare strategy with the basic package of primary health services, which include maternal and reproductive health, child health services, treatment of minor ailments, health education and follow-up on defaulters and discharged patients. It is worth noting that TB and DM care is not offered at the basic primary healthcare level, which we considered to be a plausible reason for their exclusion.

Further research exploring the perspectives of frontline healthcare workers in private for-profit health facilities, is required. It will be through understanding the perspectives from both the public and private sectors that opportunities for public-private partnership in addressing TB-DM care and control, can be explored. The plausibility of involving community-based health planning services compounds (CHPS), whose primary responsible is prevention and treatment of minor diseases, in the TB-DM care and control, is worth exploring.

## Conclusions

Our findings reiterate the need for the Ministry of Health (MOH/GHS) to prioritise and fund consistent in-service training programs for both TB and DM healthcare workers, so that healthcare workers will be better equipped to manage the growing TB-DM co-epidemic. Healthcare workers’ personal sacrifices in funding the departmental functions advantaged certain patients over others based merely on geography. Despite the inadvertent consequences of healthcare workers’ financial interventions, they are an important gesture and commendable commitment to their work specifically and patient care generally.

In line with the findings of this study and the evidence from the literature, HIV/TB integration holds a promise that an integrated screening tool for TB and DM, can be achieved. This integrated approach to screening is anticipated to further improve care and strengthen the healthcare system’s co-management of TB and DM. To achieve this integrated approach, investment in additional infrastructural and human resource support for health facilities, would be required in order to promote better collaboration with frontline healthcare workers delivering TB and DM care.

Our research offers valuable insights into the experiences of healthcare workers implementing health policy in public health facilities and serve as an important contribution to inform the implementation of WHO-Union framework in similar settings.

## Supplementary Information

**Additional file 1.**

**Additional file 2.**

## Data Availability

The datasets used and/or analysed during the current study available from the corresponding author on reasonable request.

## Abbreviations

DM: Diabetes Mellitus
DOT: Direct Observed Treatment
GHS: Ghana Health Service
HCWs: Health Care Workers
Union: International Union against Tuberculosis and Lung
LMICs: Low-and Middle-Income Countries
MOH: Ministry of Health
NR: Northern Region
NTP: National Tuberculosis Control Programme
NCDP: Non-Communicable Disease Control Program
OPD: Out- Patient Department
TB: Tuberculosis
UNION: International Union Against Tuberculosis and Lung Disease
WHO: World Health Organisation
CHPS: Community based health planning services compounds
SLB: Street-Level Bureaucrats
